# Ramadan Intermittent Fasting and Plasma Volume Variations in Individuals with Different Body Weights

**DOI:** 10.3390/medicina60071143

**Published:** 2024-07-16

**Authors:** Jihen Khalfoun, Hassane Zouhal, Raoua Triki, Wafa Jribi, Ayoub Saeidi, Abdullah Almaqhawi, Cain C. T. Clark, Ismail Laher, Abderraouf Ben Abderrahman

**Affiliations:** 1 Higher Institute of Sport and Physical Education of Ksar-Said, University of Manouba, Cité Nasr 2010, Tunisia; jihenkh9@gmail.com (J.K.); raouatriki1995@gmail.com (R.T.); abderraouf.benabderrahman@issep.uma.tn (A.B.A.); 2 M2S (Laboratoire Mouvement, Sport, Santé)—EA 1274, University of Rennes, 35000 Rennes, France; 3 Institut International des Sciences du Sport (2I2S), 35850 Irodouer, France; 4 Tunisian Research Laboratory “Sports Performance Optimization”, National Center of Medicine and Science in Sports (CNMSS) LR09SEP01, Tunis 1002, Tunisia; 5 High Institute of Sport and Physical Education Sfax, Sfax University, Sfax 3029, Tunisia; jribiwafa95@gmail.com; 6 Department of Physical Education and Sport Sciences, Faculty of Humanities and Social Sciences, University of Kurdistan, Sanandaj 6617715175, Iran; 7 Department of Family and Community Medicine, College of Medicine, King Faisal University, Al Hofuf P.O. Box 400, Saudi Arabia; aalmuqahwi@kfu.edu.sa; 8 College of Life Sciences, Birmingham City University, Birmingham B15 3TN, UK; cainctclark@gmail.com; 9 Department of Anesthesiology, Pharmacology and Therapeutics, The University of British Columbia, Vancouver, BC V6T1Z4, Canada; ismail.laher@ubc.ca

**Keywords:** obese, overweight, normal weight, ΔPV, Ramadan fasting

## Abstract

*Background*: There is increasing awareness of the physiological effects of Ramadan intermittent fasting (RIF) in obese subjects. However, there are no data on the effects of RIF on plasma volume changes (ΔPV) in individuals with different body weights. *Background and Objectives*: This study investigated the effects of RIF on ΔPV in normal-weight (NW) and overweight (OW) adult men, and adult men with obesity (OB) and severe obesity (SO). *Materials and Methods*: Thirty-two male subjects (32) were divided into four groups (*n* = 8 per group) according to their body mass index (BMI): normal weight (NW) (BMI < 25 kg/m^2^; age = 27.4 ± 3.8), overweight (OW) (BMI between 25 and 29.9 kg/m^2^; age = 26.8 ± 3.7), obese subjects (OB) (BMI between 30 and 34.9 kg/m^2^; age = 25.6 ± 2.9), and severely obesity (SO) (BMI between 35 and 40 kg/m^2^; age = 24.0 ± 4.1). Blood samples were collected for 24 h on 4 different occasions, at T0 before the start of the Ramadan month, at T1 15 days after the start of Ramadan, at T2 one day after the end of Ramadan, and at T3 on the 21st day after the end of Ramadan to determine ΔPV. All groups completed their fasting rituals for the 30 days of Ramadan. *Results*: A significant group × time effect occurred for body mass (*p* = 0.001; ES = 0.53), BMI (*p* = 0.001; ES = 0.53), and body fat percentage (*p* = 0.001; ES = 0.52). Post hoc tests indicated reductions in body mass in OB and SO at T1 (*p* = 0.03; ES = 0.21 and *p* = 0.002; ES = 0.12) and T2 (*p* = 0.03; ES = 0.31 and *p* = 0.02; ES = 0.23), reductions in BMI in OB and SO at T1 (*p* = 0.04; ES = 0.35 and *p* = 0.03; ES = 0.42) and T2 (*p* = 0.03; ES = 0.52 and *p* = 0.005; ES = 0.48), and reductions in body fat percentage only in OB AT T1 (*p* = 0.002; ES = 0.31) and T2 (*p* = 0.001; ES = 0.17). A significant group × time effect occurred for hematocrit (*p* = 0.02; ES = 0.34), hemoglobin (*p* = 0.01; ES = 0.35), and ΔPV (*p* = 0.02; ES = 0.18). Post hoc tests indicated increases in hematocrit in OB at T2 (*p* = 0.03; ES = 0.36) and hemoglobin in OB and SO at T1 (*p* = 0.03; ES = 0.35 and *p* = 0.002; ES = 0.32) and T2 (*p* = 0.003; ES = 0.21 and *p* = 0.002; ES = 0.33). There were also increases in ΔPV in OB at T1 and T2 (*p* = 0.002; ES = 0.25 and *p* = 0.003; ES = 0.22) and in SO only at T2 (*p* = 0.02; ES = 0.37). Contrast analysis indicated that NW was significantly lower than the grand mean of OW, Ob, and SO for all anthropometric and PVV variables (all *p* < 0.05). *Conclusions*: The effects of RIF on ΔPV and anthropometric characters was greater in obese individuals compared to normal-weight and overweight participants, suggesting that the improvements in body composition and ΔPV produced by RIF could positively influence obesity.

## 1. Introduction

Obesity levels continue to increase, making it a health concern worldwide [1]. The causes of obesity include uncontrolled food intake combined with a sedentary lifestyle, and obesity is associated with several complications, such as cardiovascular, metabolic, respiratory, and orthopedic hematological pathologies [2], making obesity a leading cause of mortality in both males and females [3].

Several therapeutic methods have been investigated to combat obesity and its consequences through reduced energy intake or/and increasing daily physical activity. Intermittent fasting (IF) is a frequently used to modify the eating schedule and has positively impacts obesity and health complications [4]. Ramadan intermittent fasting (RIF) is an example of a daily dietary approach, and it occurs during the ninth of the Islamic calendar, where Muslims abstain not only from food and fluids consumption but also from any type of oral intake, intravenous injection, and sexual intimacy from sunrise to sunset [5,6]. Dietary habits change during Ramadan, and many consume high-calorie meals after sunset (iftar) and another lighter meal before dawn (suhur), with a preference for foods containing more carbohydrates and starches [5]. The nutritional and chronobiologic changes during RIF offers a nutritional strategy to study the effects of intermittent fasting on the regulation of hematological changes and cardiovascular concerns in obese persons [7,8].

Previous studies have reported that RIF modulates some hematological parameters, such as increases in hemoglobin (Hb) and decreases in hematocrit (Ht) [7,9], associated with obesity [9]. Thus, we can conclude that prolonged RIF can change plasma volume variation (PVV), resulting from changes in Ht and Hb according to the equation of Dill and Costill [10]. In fact, plasma volume status is an important physiological index and a sometimes challenging area of clinical medicine. Fluid balance and blood circulation are essential in helping to maintain hemodynamic homeostasis [11].

Furthermore, abstaining from fluid intake during RIF can decrease extracellular fluid volume as well as PVV in the vascular space [12], affecting urinary sodium (Na+) loss [12,13]. Additionally, RIF also effects circulating hormones [14,15,16] and lipids [14,17], which could indirectly affect PVV.

To the best of our knowledge, there are no reports on the effects of RIF on PVV in obese subjects. Our study investigated the effect of RIF on PVV in individuals with different body weights (normal weight, overweight, obese, and individuals with severe obesity). We hypothesized that RIF reduces PVV in individuals with severe obesity, as estimated from the changes in Ht and Hb concentrations.

## 2. Materials and Methods

### 2.1. Participants

A minimum sample size of 8 participants per group was determined from an a priori statistical power analysis using G*Power (Version 3.1, University of Dusseldorf, Düsseldorf, Germany) [18]. The power analysis was computed with an assumed power at 0.9 at an alpha level of 0.01 and a very large effect size (ES) of 4 for hemoglobin, our primary outcome [19].

Thirty-two male subjects aged 20 to 40 years old participated in our study and completed the religious intermittent fasting of Ramadan during the entire month. Participants were divided into four groups (*n* = 8 per group) according to their body mass index (BMI): normal weight (NW, BMI < 25 kg/m^2^; age = 27.4 ± 3.7 yo), overweight (OW, BMI 25–29.9 kg/m^2^; age = 26.8 ± 3.7 yo), obese (OW, BMI 30–34.9 kg/m^2^; age = 25.6 ± 2.8 yo), and severe obesity (SO, BMI 35–40 kg/m^2^; age = 23.9 ± 4.1 yo) (Table 1).

An informed consent was obtained from all subjects prior to being included in the study. The inclusion criteria for participation were no cardiac, endocrine, or orthopedic diseases, no physical disabilities or cognitive impairments, no history of drug consumption before the study, and no history of smoking and alcohol use. Physical activity of the participants of <60 min/week of structured exercise was required, as assessed by the International Physical Activity Questionnaire (PAR-Q) [20]. A physician using the PAR-Q and medical health/history questionnaire evaluated all criteria. The study was approved by the Ethics Committee of the Tunisian National Center for Sports Medicine and Sciences (approval number LR09SEP01, 14 September 2020) and carried out in accordance with the Declaration of Helsinki.

### 2.2. Study Design

**Figure 1:**
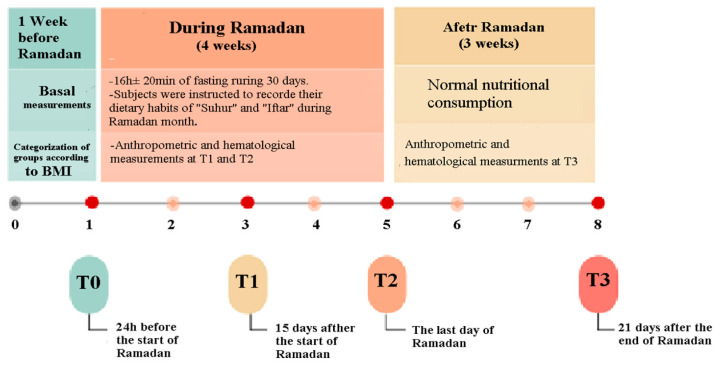
Graphical overview of study design.

This study was undertaken in Tunisia during the month of Ramadan 2019 (5 May to 3 June 2019). The duration of the fast was between 15 and 16 h per day with a mean temperature of 24 °C. All participants completed their fasting rituals from dawn until sunset for all 30 days of Ramadan. All participants were fully informed about all experimental procedures, benefits, and risks of the study. All measurements of anthropometrics and blood collection were evaluated on 4 separate occasions at the same time of day (within ~1 h): at T0 before the start of the Ramadan month, at T1 15 days after the start of Ramadan, at T2 one day after the end of Ramadan, and at T3 on the 21st day after the end of Ramadan (Figure 1). Venous blood samples (15 mL) were collected by venipuncture during the fasting state between 8 a.m. and 9 a.m. after measuring resting blood pressures first. Each sample was collected in EDTA tubes (purple) containing diaminotetracarboxylic acid.

#### 2.2.1. Dietary Habits Control

The nutritionist of the sports center conducted dietary surveys to record the nutritional intake of the participants. Every participant completed a two-day food diary survey before each of the blood samplings. The energy value of the nutrients consumed was calculated using the method of McCance (Table 2).

#### 2.2.2. Anthropometric Measurements

Body parameters were measured by the same examiner in accordance with the positions and techniques established by the International Biological Program [21]. Body weight was measured with a precision of 0.1 kg using a digital scale (Lumbar, China), with the participants not wearing shoes and dressed in light clothing. Height was measured with a precision of 0.1 cm using a medical sampler (Race Industrialization, China), and the body mass index (BMI) determined by dividing the weight (kg) by the square of the height (m) (kg/m^2^). The body fat percentage was determined using a multi-frequency bioelectrical impedance device (In body 720, Republic of Korea). The waist circumference was measured to the nearest 0.5 cm at the horizontal plane midway between the lowest ribs and the iliac crest, while the hips circumference was measured at the largest circumference around the buttocks, then the waist-to-hip ratio (WHR) was calculated using the following formula: WHR = waist circumference/hip circumference.

#### 2.2.3. Blood Sampling and Analysis

Participants were instructed to follow some instructions before each blood sample was drawn:

1. Avoid any type of medications or supplements;
2. Avoid any physical activity of a higher intensity for at least 72 h before the blood sample;
3. Follow the same alimentation 48 h before each blood sample.

Samples were collected in two pre-cooled 4.9-mL EDTA vacuettes (Klap activator model), and whole blood samples were analyzed for a complete blood count using an automated cell counter. The concentration of hemoglobin (*Hb*) and the percentage of hematocrit (*Ht*) for each sample were measured to determine variations in plasma volume. The variation in plasma volume during the different periods of the experiment was determined using the Dill and Costill method [10]:

$$PVV\left(\%\right)=100\times \frac{Hb\left(n\right)}{Hb\left(n+1\right)}\left(\times \frac{\left(1-Ht\left(n+1\right)\times 10^{-2}\right)}{\left(1-Ht\left(n\right)\times 10^{-2}\right)}\right)-100$$

*PVV*: plasma volume variation expressed in % PVV, n: value measured at one of the measurement periods, n+1: value following the previous period, *Ht*: hematocrit (%), *Hb*: hemoglobin (g/dL).

### 2.3. Statistical Analysis

Statistical Package for Social Science (SPSS) version 16.0 (SPSS Inc., Armonk, NY, USA) and R [22]. R: A Language and environment for statistical computing. Computer software retrieved from https://cran.r-project.org/, access on 15 January 2020. were used for the statistical analysis. Data are presented as mean ± SD (standard deviation). The normality of the data distribution was confirmed using the Kolmogorov–Smirnov test, while the two-way analysis of variance (ANOVA) for repeated measures (time × groups) was used to analyze differences within and between groups. If a statistically significant interaction or main effect was found, Newman–Keul’s post hoc tests were calculated to identify specific differences. The effect size (ES) was determined for all comparisons made by converting partial eta-squared values into Cohen’s D-values using the ANOVA output. In addition, the within-group ES was calculated using the following equation: ES = (post mean − pre mean)/SD [23]. Measures of ES were considered trivial (<0.2), small (0.2–0.6), moderate (0.6–1.2), large (1.2–2, 0), and very large (2.0–4.0) [24].

We also conducted a pre-planned contrast analysis, wherein we hypothesized that NW would outperform any overweight condition for all anthropometric and PVV variables. Accordingly, we compared the NW condition vs. overweight, obesity, and severe obesity (coded as OW, OB, and SO, respectively). This approach compared the NW condition vs. the grand mean of the specified contrasts. Notwithstanding the utility of post hoc testing, it does not inherently yield a sufficient insight into specific response patterns, whilst contrast analysis allows theory-driven expectations to be tested directly against empirically derived group or cells means, which in this instance is NW vs. any overweight/obesity group [25].

The percentage of variation (%) in the anthropometric measurements was measured using the formula: $\left(\frac{final\;value-initial\;value}{initial\;value}\right)\times 10$.

Additionally, correlations between the plasma volume variation and the variation in anthropometric variables during the experiment were assessed using Pearson’s product-moment correlation coefficient (r). Statistical significance was accepted, a priori, at *p* < 0.05.

## 3. Results

All participants in this study reported a 100% adherence to the study procedures. The anthropometric measurements and the plasma volume variation values among the four groups of participants with different weights were analyzed.

### 3.1. Anthropometric Measurements

The mean value ± SD of the anthropometric parameters among the groups during the four phases are presented in Table 3. There was a significant group × time effect for body mass (*p* = 0.001; ES = 0.53), BMI (*p* = 0.001; ES = 0.53), and body fat percentage (*p* = 0.001; ES = 0.52). A post hoc test indicated significant reductions in body mass for OB and SO at T1 (*p* = 0.03; ES = 0.21 and *p* = 0.002; ES = 0.12) and T2 (*p* = 0.03; ES = 0.31 and *p* = 0.02; ES = 0.23), with significant reductions in BMI among OB and SO at T1 (*p* = 0.04; ES = 0.35 and *p* = 0.03; ES = 0.42) and T2 (*p* = 0.03; ES = 0.52 and *p* = 0.005; ES = 0.48) and in body fat percentage occurring only in OB at T1(*p* = 0.002; ES = 0.31) and T2 (*p* = 0.001; ES = 0.17).

A contrast analysis indicated that body mass in the NW group was lower than the grand means of all OW/OB/SO conditions at every time point (T0: −538.25, T1: −37.34, T2: −36.79, T3: −37.05, all *p* < 0.001). The BMI of the NW group was lower than the grand mean of all OW/OB/SO conditions at all time points (T0: −11.92, T1: −11.63, T2: −11.46, T3: −11.54, all *p* < 0.001). The body fat percentage of the NW group was lower than the grand mean of all OW/OB/SO conditions at every time point (T0: −15.12, T1: −14.76, T2: −14.56, T3: −14.65, all *p* < 0.001). The weight of the NW group was lower than the grand mean of all OW/OB/SO conditions at every time point (T0: −30.52, T1: −30.28, T2: −29.95, T3: −30.08, all *p* < 0.001). The hip measurements of the NW group were lower than the grand mean of all OW/OB/SO conditions at every time point (T0: −18.28, T1: −18.03, T2: −18.08, T3: −18.1, all *p* < 0.001), and finally, the W/H ratio of the NW group was lower than the grand mean of all OW/Ob/SO conditions at every time point (T0: −0.172, T1: −0.168, T2: −0.167, T3: −0.169, *p* = 0.03, 0.02, 0.02, and 0.01, respectively).

### 3.2. Plasma Volume Variation

**Figure 2:**
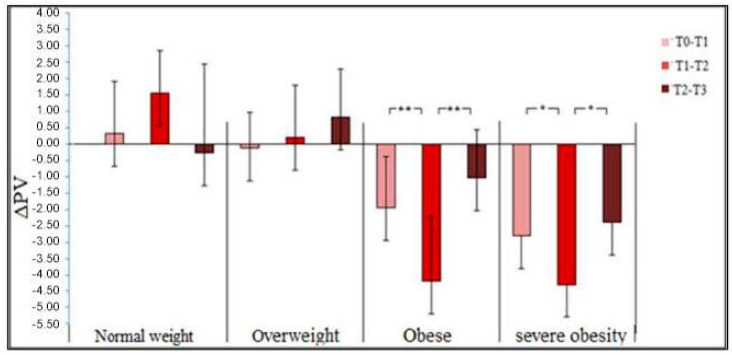
Plasma volume variations in groups with different body weights. **: *p* < 0.01; *: *p* < 0.05.

The mean values of the hemoglobin concentrations and variations in hematocrit and plasma volume in the different groups during the four phases of the study are shown in Table 4 and Figure 2. There were group × time effects for hematocrit (*p* = 0.02; ES = 0.34), hemoglobin (*p* = 0.01; ES = 0.35), and ΔPV (*p* = 0.02; ES = 0.18). A post hoc test indicated increases in Ht in the OB group at T2 (*p* = 0.03; ES = 0.36), increases in hemoglobin in the OB and SO groups at T1 (*p* = 0.03; ES = 0.35 and *p* = 0.002; ES = 0.32) and T2 (*p* = 0.003; ES = 0.21 and *p* = 0.002; ES = 0.33), and increases in ΔPV in the OB group at T1 and T2 (*p* = 0.002; ES = 0.25 and *p* = 0.003; ES = 0.22) and in the SO group only at T2 (*p* = 0.02; ES = 0.37).

A contrast analysis indicated lower Ht values in the NW group than the grand mean of all OW/OB/SO groups at all time points (T0: −5.28, T1: −6.14, T2: −6.37, T3: −2.27, all *p* < 0.001). The levels of HB were lower in the NW group than the grand mean of all OW/OB/SO groups at all time points (T0: −2.2, T1: −2.28, T2: −2.58, T3: −2.26, all *p* < 0.001). The PVV value in the NW group was different than the grand mean of all OW/OB/SO conditions at T0–T1 (1.95, *p* = 0.02) and T0–T2 (4.32, *p* < 0.001), but not T2–T3 (0.62, *p* = 0.51).

### 3.3. Correlations of Variables with Body Weights

The relationships between percent changes in anthropometric measurements and those of plasma volume variations in the different groups are shown in Table 5, indicating relationships only in the obese group for some anthropometric parameters (body mass, BMI, and body fat percentage) and ΔPV, respectively (r = −0.479, *p* = 0.018; r = −0.479, *p* = 0.018 and r = −0.482, *p* = 0.017).

## 4. Discussion

We investigated the effects of Ramadan intermittent fasting on plasma volume variations in subjects with different body weights. Hematocrit values increased in obese subjects at T2, with increases in hemoglobin levels in obese and severely obese groups at T1 and T2. We measured greater changes in PV during Ramadan only in obese and severely obese participants. Anthropometric parameters were also measured due to their correlation with obesity; there were decreases in body composition in obese and severely obese subjects, but with no measurable effects of RIF on the anthropometric characteristics of normal-weight and overweight subjects, as also reported in other studies [7,25,26]. Other investigations reported that RIF lowers the visceral adiposity and body weight in overweight and obese individuals [27,28]. Another study reported increases in weight, BMI, and body fat percentage in normal-weight and obese subjects by the end of Ramadan [29]. Thus, the decreases in body weight we recorded in the obese subjects may be due to significant dehydration during Ramadan, as they have a greater volume of body water compared to normal-weight subjects [15]. Our study also indicates increases in hemoglobin concentrations and decreases in PVV in persons with obesity and severe obesity after Ramadan, with a trend for increasing hematocrit levels only in obese persons after Ramadan fasting.

No previous study has examined the effects of RIF on plasma volume variation as a function of body weight; in fact, plasma volume can vary based on body weight, lean body mass, fat percentage, basal metabolic rate, and nutritional condition [30]. Thus, it is important to investigate the effect of intermittent fasting on PVV, since it is an index of a good physiological balance between the intake of food and liquids versus output through respiration, urine, feces, and skin [31]. 

Previous studies have examined the effect of RIF on Ht and Hb variation in healthy subjects, with some reporting significant increases [5,29], significant decreases [32,33,34], or no change [6]. Ünalacak et al. [7] reported that RIF did not change hematocrit and hemoglobin concentrations in normal-weight subjects.

The decreases in plasma volume variation in obese participants in our study could be related to consecutive daytime abstentions from fluids, salts, and other foods during RIF, which can increase plasma concentration and decrease plasma volume [35] and elevate levels of plasma sodium and plasma chloride [36]. Thus, the quantity of body water in obese persons compared to normal-weight subjects [15] could decrease plasma volume variations due to dehydration.

In fact, our measurements were conducted in Tunisia during the month of Ramadan 2019, where the fasting hours were between 15 to 16 per day and the mean temperature was 24 °C. As we know, fasting hours during Ramadan change according to geographic location and season, as it follows the sunrise and sunset moments; additionally, day temperature plays an important role in dehydration during fasting. Therefore, we cannot generalize our results for Ramadan fasting under different conditions (fasting hours and temperature).

It is important also to compare Ramadan fasting with other types of intermittent fasting, such as alternate day fasting and time-restrictive feeding. Future studies should also conduct research among females to study the effect of menstruation during Ramadan intermittent fasting on plasma volume variation and body composition.

## 5. Conclusions

Our results suggested that Ramadan intermittent fasting has a positive effect on ΔPV and anthropometric measurements among subjects fasting during this month. This improvement was detected among obese and severely obese persons, where the plasma volume was higher during the month of Ramadan, associated with an increasing of hemoglobin concentration. This hypervolemia can be an index for a larger vascular volume, a greater cardiac filling, a better cardiovascular stability, and a maximal cardiac output. Therefore, RIF regimes seem to be adjutant strategies for an increasing weight among patients with obesity and improving the hematological system to avoid cardiovascular diseases.

## Figures and Tables

**Table 1:** Anthropometric and body composition characteristics (means ± SD) among different weight statuses at baseline.

Variables	NW	OW	Ob	SO
Age (years)	27.4 ± 3.7	26.8 ± 3.7	25.6 ± 2.8	23.9 ± 4.1
Height (cm)	178.1 ± 3.6	179.8 ± 3.9	178.6 ± 2.0	177.1 ± 2.2
Body mass (kg)	65.5 ± 5.2	93.3 ± 5.1	106.0 ± 2.7	112.3 ± 4.3
BMI (kg/m^2^)	20.6 ± 1.6	28.8 ± 0.7	33.2 ± 0.4	35.7 ± 0.5
Body fat (%)	14.6 ± 1.6	25.1 ± 1.2	30.5 ± 1.1	33.7 ± 1.2
Waist (cm)	68.5 ± 3.4	80.3 ± 2.3	105.0 ± 5.2	112.0 ± 1.4
Hip (cm)	85.6 ± 1.4	93.6 ± 1.6	112.2 ± 5.0	112.4 ± 2.1
WHR	0.8 ± 0.0	0.8 ± 0.0	0.9 ± 0.0	0.9 ± 0.0

Body mass index; WHR: waist-to-hip ratio; NW: normal weight; OW: overweight; Ob: obese; SO: severe obesity.

**Table 2:** Macronutrient values (means ± SD) at T0, T1, T2, and T3. Data are expressed as mean ± SD.

Protein (% kcal)		
Groups	T0	T1
NW	18 ± 1	19 ± 7
OW	17 ± 3	20 ± 5
Ob	18 ± 1	20 ± 3
SO	18 ± 2	20 ± 5
Groups	T0	T1
Hip (cm)	85.6 ± 1.4	93.6 ± 1.6
Carbohydrates (% kcal)		
Groups	T0	T1
NW	47 ± 8	47 ± 5
OW	46 ± 2	45 ± 4
Ob	46 ± 8	46 ± 8
SO	47 ± 7	46 ± 8
Lipids (% kcal)		
Groups	T0	T1
NW	35 ± 4	35 ± 4
OW	37 ± 6	35 ± 4
Ob	36 ± 4	36 ± 5
SO	36 ± 4	36 ± 4

NW: normal weight; OW: overweight; Ob: obese; SO: severe obesity; T0: 24 h before start of Ramadan (basal measurements); T1: 15th day of Ramadan; T2: day after end of Ramadan; T3: 21 days after end of Ramadan.

**Table 3:** Anthropometric and body composition characteristics (means ± SD) measured at T0, T1, T2, and T3 among all groups.

	Group	Phases				*p*-Values (ES)		
		T0	T1	T2	T3	Time	Group	Group × Time
Body mass (kg)	NW	65.5 ± 5.2	65.6 ± 5.1	65.8 ± 4.8	65.8 ± 4.7	0.001 (0.95)	0.001 (0.77)	0.001 (0.53)
	OW	93.3 ± 5.1	93.2 ± 5.1	93.3 ± 5.1	93.3 ± 5.0			
	OB	106.0 ± 2.7	104.6 ± 2.7 *	103.7 ± 3.0 **	104.1 ± 3.0			
	SO	112.3 ± 4.3	111.4 ± 4.3 *	111.1 ± 4.5 *	111.2 ± 4.5			
BMI (kg/m^2^)	NW	20.6 ± 1.6	20.7 ± 1.6	20.7 ± 1.5	20.7 ± 1.5	0.001 (0.98)	0.001 (0.77)	0.001 (0.53)
	OW	28.8 ± 0.7	28.7 ± 0.7	28.8 ± 0.7	28.8 ± 0.7			
	OB	33.2 ± 0.4	32.8 ± 0.5 *	32.5 ± 0.6 *	32.6 ± 0.6			
	SO	35.7 ± 0.5	35.5 ± 0.5 *	35.4 ± 0.5 **	35.4 ± 0.5			
Body fat percentage (%)	NW	14.6 ± 1.6	14.7 ± 1.5	14.7 ± 1.4	14.7 ± 1.4	0.001 (0.97)	0.001 (0.77)	0.001 (0.52)
	OW	25.1 ± 1.2	25.04 ± 1.2	25.09 ± 1.2	25.11 ± 1.2			
	OB	30.5 ± 1.1	30.0 ± 1.1 **	29.6 ± 1.3 ***	29.8 ± 1.3			
	SO	33.7 ± 1.2	33.4 ± 1.1	33.3 ± 1.2	33.3 ± 1.3			
Waist (cm)	NW	68.5 ± 3.4	68.4 ± 3.2	68.6 ± 3.3	68.5 ± 3.3	0.13 (0.28)	0.001 (0.97)	0.74 (0.28)
	OW	80.3 ± 2.3	79.66 ± 3.4	79.7 ± 3.4	79.8 ± 3.5			
	OB	105.0 ± 5.2	104.8 ± 5.1	104.6 ± 5.1	104.6 ± 5.0			
	SO	112.0 ± 1.4	111.9 ± 1.3	111.7 ± 1.4	111.7 ± 1.4			
Hip (cm)	NW	85.6 ± 1.4	85.7 ± 1.5	85.5 ± 1.4	85.6 ± 1.3	0.51 (0.62)	0.001 (0.96)	0.59 (0.51)
	OW	93.6 ± 1.6	93.7 ± 1.4	93.6 ± 1.7	93.7 ± 1.7			
	OB	112.2 ± 5.0	112.1 ± 5.0	112.0 ± 5.1	112.0 ± 5.2			
	SO	112.4 ± 2.2	111.9 ± 1.1	111.8 ± 1.5	111.9 ± 1.3			
WHR	NW	0.8 ± 0.0	0.7 ± 0.0	0.8 ± 0.0	0.8 ± 0.0	0.53 (0.63)	0.001 (0.93)	0.54 (0.81)
	OW	0.8 ± 0.0	0.8 ± 0.0	0.8 ± 0.0	0.8 ± 0.0			
	OB	0.9 ± 0.0	0.9 ± 0.0	0.9 ± 0.0	0.9 ± 0.0			
	SO	0.9 ± 0.0	1.0 ± 0.0	0.9 ± 0.0	0.9 ± 0.0			

BMI: body mass index; WHR: waist-to-hip ratio; NW: normal weight; OW: overweight; OB: obese; SO: severe obesity; T0: 24 h before start of Ramadan (basal measurements); T1: 15th day of Ramadan; T2: day after end of Ramadan; T3: 21 days after end of Ramadan; ES: effect size; ***: *p* < 0.001; **: *p* < 0.01; *: *p* < 0.05.

**Table 4:** Hematocrit, hemoglobin, and plasma volume variation (means ± SD) of all groups measured at four time points.

Variable	Group	Phases				*p*-Values (ES)		
		T0	T1	T2	T3	Time	Group	Group × Time
Ht (%)	NW	42.2 ± 0.3	41.8 ± 0.7	41.7 ± 0.7	42.4 ± 0.3	0.06 (0.32)	0.001 (0.62)	0.02 (0.34)
	OW	46.2 ± 3.5	46.7 ± 3.7	46.4 ± 3.7	46.1 ± 3.6			
	OB	49.3 ± 2.4	49.8 ± 2.3	50.4 ± 2.4 *	49.8 ± 2.5			
	SO	49.1 ± 2.0	49.5 ± 2.1	49.7 ± 2.2	49.7 ± 2.3			
[Hb] (g·100 mL^−1^)	NW	14.3 ± 0.2	14.4 ± 0.3	14.2 ± 0.1	14.3 ± 0.1	0.04 (0.35)	0.001 (0.69)	0.01 (0.35)
	OW	15.9 ± 1.3	15.8 ± 1.3	15.8 ± 1.3	15.8 ± 1.0			
	OB	16.6 ± 0.9	16.7 ± 0.9 *	16.9 ± 0.9 **	16.6 ± 0.9			
	SO	17.2 ± 1.1	17.5 ± 1.2 **	17.7 ± 1.0 **	17.4 ± 0.9			
		T0–T1		T1–T2	T2–T3			
ΔPV (%)	NW	0.3 ± 1.9		1.5 ± 3.0	−0.2 ± 1.1	0.04 (0.37)	0.03 (0.42)	0.02 (0.18)
	OW	−0.1 ± 2.4		0.1 ± 2.0	0.8 ± 3.2			
	OB	−1.9 ± 1.0		−4.1 ± 1.7 **	−1.0 ± 2.4 **			
	SO	−2.8 ± 3.4		−4.2 ± 4.2	−2.4 ± 3.1 *			

NW: normal weight; OW: overweight; OB: obese; SO: severe obesity; T0: 24 h before start of Ramadan (basal measurements); T1: 15th day of Ramadan; T2: day after end of Ramadan; T3: 21 days after end of Ramadan; ΔPV: plasma volume variation; [Hb]: hemoglobin concentration; Ht: hematocrit; ES: effect size; **: *p* < 0.01; *: *p* < 0.05.

**Table 5:** Relationships between % changes in anthropometric measurements and PVV.

Variable	Group	Δ Body Mass (%)	Δ BMI (%)	Δ BFP (%)	Δ Waist (%)	Δ Hip (%)	Δ WHR (%)
ΔPV (%)	NW	r = 0.258 *p* = 0.217	r = 0.268 *p* = 0.206	r = 0.25 *p* = 0.238	r = −0.018 *p* = 0.933	r = −0.075 *p* = 0.729	r = 0.065 *p* = 0.762
	OW	r = −0.225 *p* = 0.291	r = −0.225 *p* = 0.291	r = −0.219 *p* = 0.304	r = 0.172 *p* = 0.421	r = 0.139 *p* = 0.517	r = 0.113 *p* = 0.598
	Ob	r = −0.479 * p = 0.018	r = −0.479 * p = 0.018	r = −0.482 * p = 0.017	r = −0.33 *p* = 0.879	r = −0.296 *p*=0.161	r = 0.087 *p* = 0.686
	SO	r = −0.088 *p* = 0.683	r = −0.086 *p* = 0.694	r = −0.083 *p* = 0.701	r = −0.121 *p* = 0.574	r = −0.178 *p* = 0.404	r = 0.161 *p* = 0.453

BMI: body mass index; BFP: body fat percentage; WHR: waist-to-hip ratio; NW: normal weight; OW: overweight; Ob: obese; SO: severe obesity; ΔPV: plasma volume variation; *: *p* < 0.05.

## Data Availability

Data are available upon request from the corresponding author.
